# Correlation Between Body Mass Index and Anterolateral Thigh Flap Thickness: A Retrospective Study From a Single Center in China

**DOI:** 10.3389/fsurg.2021.748799

**Published:** 2021-10-11

**Authors:** Jianxin Yin, Lei Wang, Gongxin Yang, Xingjun Qin, Ping Xiong

**Affiliations:** ^1^Department of Ultrasound, Shanghai Ninth People's Hospital, Shanghai Jiaotong University School of Medicine, Shanghai, China; ^2^Guangzhou Key Laboratory of Basic and Applied Research of Oral Regenerative Medicine, Department of Oral and Maxillofacial Surgery, Affiliated Stomatology Hospital of Guangzhou Medical University, Guangzhou, China; ^3^Department of Radiology, Shanghai Ninth People's Hospital, Shanghai Jiaotong University School of Medicine, Shanghai, China; ^4^Department of Oral and Maxillofacial–Head and Neck Oncology, Shanghai Ninth People's Hospital, College of Stomatology, Shanghai Jiao Tong University School of Medicine, Shanghai, China; ^5^National Clinical Research Center for Oral Diseases, Shanghai, China; ^6^Shanghai Key Laboratory of Stomatology and Shanghai Research Institute of Stomatology, Shanghai, China

**Keywords:** body mass index, anterolateral thigh flap, thickness, retrospective study, ultrasound

## Abstract

**Background:** During repair of oral and maxillofacial soft tissue defects, organ function is largely related to the amount of thickness of the flap. However, there are few studies on the influencing factors of the thickness of the flap. In this retrospective study, we aim to explore the correlation between body mass index (BMI) and anterolateral thigh (ALT) flap thickness by computed tomography (CT) and ultrasound and provide guidance for evaluating the ALT flap thickness before surgery.

**Methods:** We selected three points A, B, and C on ALT flap and two skilled clinicians measured the thickness of these points. Age and gender as covariates and evaluated by the Chi-square analysis. Inter-group differences between the two BMI groups were examined by the student *t* test. Intra-group differences within each BMI group were tested by ANOVA. Linear regression analysis was performed to examine the relationship between BMI and ALT flap thickness.

**Results:** One hundred sixty patients measured by CT were included in this study, and the ALT flap thickness measured by CT were 8.96 mm and 11.00 mm (*P* < 0.0001, *t* test) at point B in groups with BMI<24.0 and BMI≥24.0, respectively. The thicknesses at points A, B, and C were significantly correlated with the BMI (P < 0.001, correlation analysis, r = 0.462, 0.372, and 0.349 at the points A, B, and C, retrospectively, Pearson test).

**Conclusion:** There was a significant correlation between the ALT flap thickness and BMI. A higher BMI was correlated with a thicker ALT flap.

## Introduction

Repair and reconstruction of soft tissue defects have always been a difficult but important process during the oral and maxillofacial operation (1). Selection of the appropriate type and thickness of flap is crucial in achieving satisfactory outcomes. The flap should not only cover the defective wound but also restore the organ functions, such as tongue movements, tongue–palate contact, eating, and pronunciation. The restoration of organ functions is largely directly related to the amount of tissue and thickness of the flap (2). The anterolateral thigh (ALT) flap is the most commonly used flap to repair soft tissue defects in the oral and maxillofacial area due to its large tissue volume, long vascular pedicle, relatively simple preparation, and wide range of applications (2–6). The function of the repaired defective tissue largely depends on the intra-operative selection of the ALT flap with appropriate thickness.

In the clinic, the thickness of an ALT flap is measured from the skin surface to the fascia lata in the thigh. For thick flaps, clinicians often thinned the flap intra-operatively (7, 8). However, no matter whether intra-operative immediate or delayed thinning was performed, or whether the thinning was performed before or after the dissection or after vascular anastomosis, the procedures usually took a significant amount of time and effort, which prolonged the operative duration and increased the risk of damage to the perforating vessels (9). This could lead to partial or complete necrosis of the flap, resulting in surgical failure (10–12). Affected patients can experience significant physical trauma and pain, as well as additional financial losses. The thickness of the ALT flap varies significantly among different individuals. There is no reliable method to perform a pre-operative evaluation. Therefore, it is necessary and urgently required to determine an effective way to identify the factors that are associated with the flap thickness among different individuals.

Several previous studies have reported a correlation between the ALT flaps and body mass index (BMI). Yu found a satisfactory correlation between the thickness of the ALT flap and BMI in 68 well-prepared flaps (13). Another study also showed strong correlations between BMI and the thickness of different types of flaps (forearm radial flap, ALT flap, and lateral peroneal flap), with the most significant correlation observed between the ALT flap and BMI (14). However, this study used ultrasound measurements to obtain the data. Although ultrasound testing is commonly used in image analysis and has the advantages of being inexpensive and non-invasive, the results of the ultrasound are highly operator dependent and easily affected by multiple factors. For example, different operators may apply different pressures on the probes while measuring the thickness of the flap. This can lead to different measurements, which can affect the stability and reproducibility of the results.

To determine the correlation between BMI and ALT flap thickness more objectively and accurately, we performed a retrospective study and analyzed the ALT thickness shown on computed tomography (CT) in patients with different BMI values. We aimed to determine the relationship between BMI and ALT flap thickness.

## Methods

### Study Design and Participant Selection

we performed a retrospective study at the ninth people's Hospital, Shanghai Jiaotong University School of Medicine, Shanghai, China. The study was approved by the hospital ethics committee (SH9H-2021-T19-2). The requirement for obtaining informed consent from patients was waived.

Patient inclusion criteria were as follows: (1), age 18–80 years; (2), complete data of lower extremity CT angiography or CT venography examination at our hospital between January 2018 and October 2020; (3), with height and weight measurements in the medical record. Exclusion criteria were as follows: 1) lower extremity deformity due to surgical or other treatments; (2) lower extremity atrophy due to congenital or developmental disorders.

### Outcome Measurements

The imaging data were obtained from the radiology department (PACS system) and the ultrasound diagnostic department. These images were measured by two experienced clinicians, with adequate educational background and clinical practice skills, as well as in-depth understanding of the anatomy of the ALT flap. The measurement standards and methods were standardized before the initiation of the study to minimize the influence of individual performances. All patient data were stored in an encrypted network drive and were accessible only to the research personnel who were involved in the present study.

**Figure 1:**
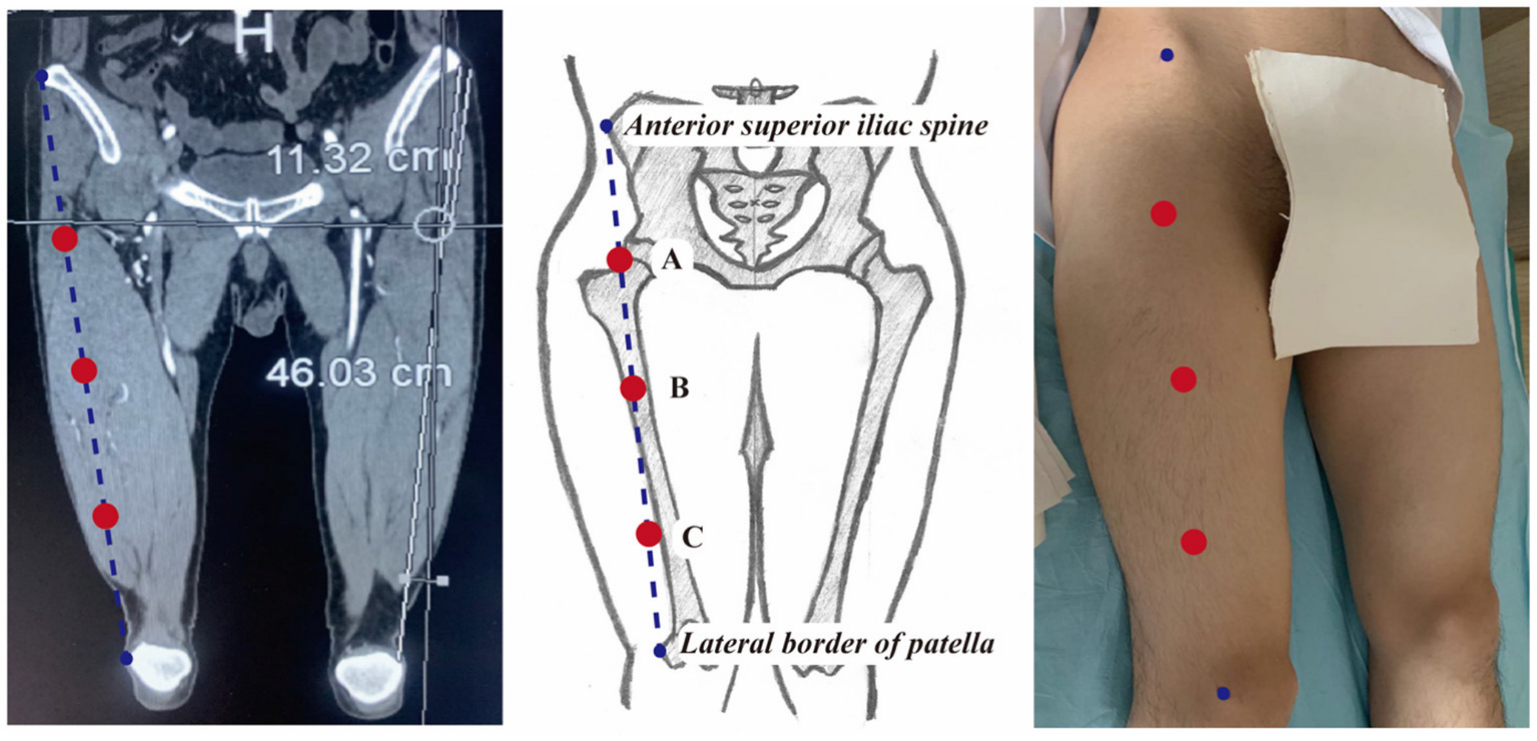
Schematic diagram of the selected marker points to measure the ALT flap thickness.

The thickness of the ALT flap was defined as the vertical distance from the fascia at the junction of the rectus femoris and the superficial surface of the vastus lateralis to the skin surface. The detailed measurement steps were as follows: (1) a straight line was drawn to connect the anterior superior iliac spine and the lateral superior patellar angle. This line was roughly overlapped with the surface projection of the muscular space between the rectus femoris and vastus lateralis; (2) the midpoint of this line was defined as point B; (3) the midpoint between the anterior superior iliac spine and point B was defined as point A; (4) the midpoint between point B and the lateral superior patellar angle was defined as point C; (5) the thickness of the flaps was measured in the cross-sectional views at points A, B, and C, separately (Figure 1).

**Figure 2:**
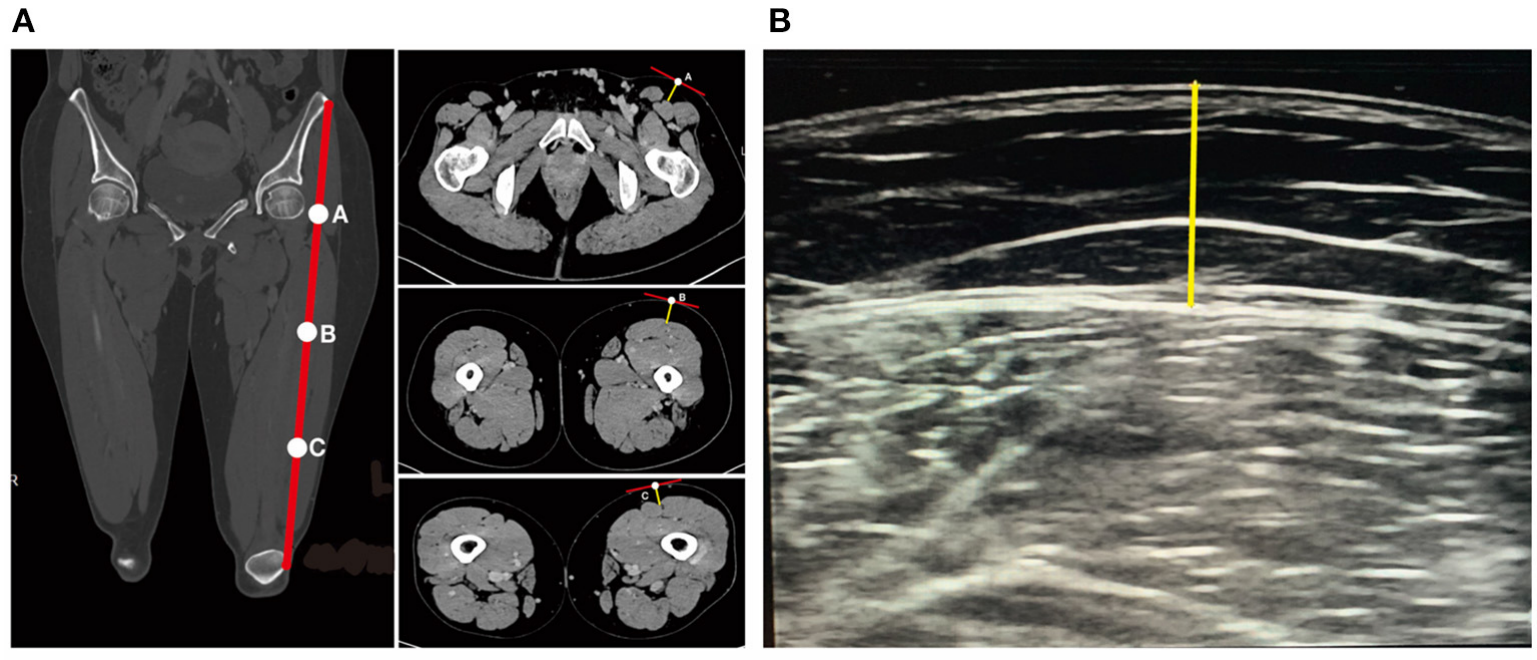
Measurements of ALT flap thickness by CT or ultrasound. **(A)** Measurement of ALT flap thickness by CT. Yellow straight line indicates the thickness of ALT flap. **(B)** Measurement of ALT flap thickness by ultrasound. Yellow straight line indicates the thickness of ALT flap.

All image analyses were performed in the software Mimics (version 21.0, Materialise NV, Belgium). CT images were imported into the Mimics software. The vertical distances from the skin surface to the fascia lata at the junction of the rectus femoris and the superficial surface of vastus lateralis in the cross-sectional views at points A, B, and C were measured (Figure 2A). Same measurements were performed on the images obtained by the color Doppler ultrasound (Figure 2B).

The main outcome was the flap thickness at point B measured by the CT scan. The secondary outcomes were the flap thickness at points A and C measured by the CT scan and the flap thickness at points A, B, and C measured by ultrasound. Additional demographic data, including age and gender, were obtained from the medical record.

### Statistical Analysis

In calculating the sample size, with β = 0.8 and α = 0.05 (two-tailed test), at least 114 study participants were required (PASS Sample Size Software 15.0, NCSS LLC, Kaysville, Utah, USA).

The Chinese classification of BMI is as follows: BMI < 18.5 (underweight), 18.5 ≤ BMI <24.0 (normal), 24.0 ≤ BMI <28.0 (overweight), and BMI ≥ 28.0 (overweight) groups. Since our study identified very few participants with BMI < 18.5 and ≥ 28.0, we assigned all study participants into two groups, BMI <24.0 and BMI ≥ 24.0.

Differences in the age and gender between the two BMI groups were evaluated by the Chi-square analysis. If *p* value was < 0.05, propensity score matching was used to ensure comparability between the two groups. Inter-group differences between the two BMI groups were examined by the student *t* test. Intra-group differences within each BMI group were tested by ANOVA. *P* < 0.05 was considered statistically significant. Linear regression analysis was performed to examine the relationship between BMI and ALT flap thickness at points A, B, and C. *P* < 0.05 suggested a statistically significant correlation.

## Results

### Baseline Characteristics of the Study Participants

A total of 160 study participants with CT images of the lower extremity were identified during the study period, with 85 participants (53.1%) in the BMI < 24.0 group and 75 participants (46.9%) in the BMI ≥ 24.0 group. Age and gender were comparable between these two groups. To study the potential differences between the CT and ultrasound measurements, we retrospectively collected additional 50 participants with ultrasound results of the lower extremity. Of these 50 participants, 31 (62.0%) were in the BMI < 24.0 group and 19 (38.0%) were in the BMI ≥ 24.0 group. Age and gender were also comparable between these two groups (Table 1).

**Table 1:** Demographic characteristics of the study participants who received CT or ultrasound examination (P was calculated by the Chi-square test).

**CT**			
	**BMI < 24.0 (*****N*** **= 85)**	**BMI ≥ 24.0 (*****N*** **= 75)**	***p*** **-value**
**Number (percentage)**			
**Gender**			0.9053
Male	45 (52.9)	39 (52.0)	
Female	40 (47.1)	36 (48.0)	
**Age**			0.9184
≤ 30	1 (1.2)	1 (1.3)	
31 ~ 50	20 (23.5)	19 (25.3)	
51 ~ 70	48 (56.5)	44 (58.7)	
>70	16 (18.8)	11 (14.7)	
**B ultrasound**			
	**BMI < 24.0 (*****N*** **= 31)**	**BMI ≥ 24.0 (*****N*** **= 19)**	***p*** **-value**
**Number (percentage)**			
**Gender**			0.2163
Male	14 (45.2)	12 (63.2)	
Female	17 (54.8)	7 (36.8)	
**Age**			0.2162
≤ 30	0 (0)	0 (0)	
31 ~ 50	10 (32.3)	2 (10.5)	
51 ~ 70	17 (54.8)	14 (73.7)	
>70	4 (12.9)	3 (15.8)	

### Differences in the ALT Flap Thickness Between Different Measuring Points and Different Groups

**Figure 3:**
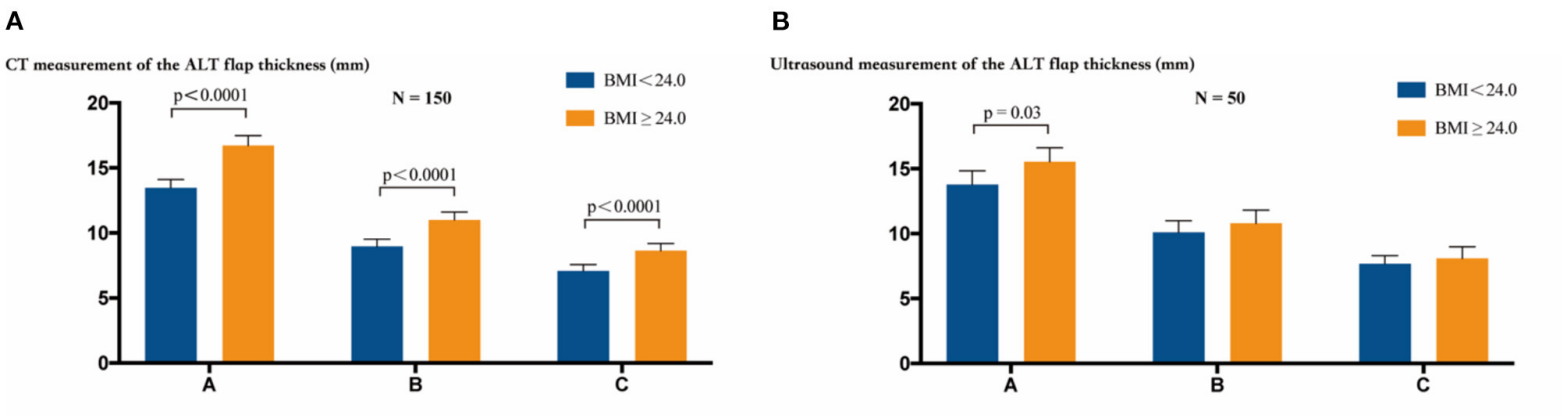
The thickness of the ALT flap was measured at points A, B, and C by CT or ultrasound examination. **(A)** CT measurement of the ALT flap thickness. Inter-group comparisons showed a statistically significant difference in the thickness at points A, B, and C between the two groups with BMI < 24.0 and BMI ≥ 24.0 (*P* < 0.0001, *t* test). Intra-group comparisons showed statistically significant differences in the thickness at points A, B, and C (*P* < 0.0001, ANOVA) within the group of BMI < 24.0 or BMI ≥ 24.0. **(B)** Ultrasound measurement of the thickness of the ALT flap. Inter-group comparisons showed a statistically significant difference only at point A between the groups with BMI < 24.0 and BMI ≥ 24.0 (*P* = 0.03, *t* test). Intra-group comparison showed statistically significant differences in the thickness at points A, B, and C (*P* < 0.0001, ANOVA) within the group of BMI < 24.0 or BMI ≥ 24.0. The bars show the mean values with 95% confidence intervals.

In these 160 study participants with complete CT data, the measurements for the ALT flap thickness were 8.96 mm and 11.00 mm (*P* < 0.0001, *t* test) at point B, 13.48 mm and 16.73 mm (*P* < 0.0001, *t* test) at point A, and 7.078 mm and 8.849 mm (*P* < 0.0001, *t* test) at point C in groups with BMI < 24.0 and BMI ≥ 24.0, respectively. Within each group (BMI < 24.0 or BMI ≥ 24.0), the flap thicknesses at points A, B, and C showed a decrease, with statistically significant differences among each measurement (*P* < 0.0001, ANOVA, Figure 3A).

In the 50 study participants with ultrasound measurements, the measurements of the ALT flap thickness were 10.10 mm and 10.79 mm (*P* = 0.32, *t* test) at point B, 13.80 mm and 15.54 mm (*P* = 0.003, *t* test) at point A, and 7.68 mm and 8.09 mm (*P* = 0.44, *t* test) at point C in groups with BMI < 24.0 and BMI ≥ 24.0, respectively. Within each group (BMI < 24.0 or BMI ≥ 24.0), the flap thicknesses at points A, B, and C showed a decrease, with statistically significant differences among each measurement (*P* < 0.0001, ANOVA, Figure 3B).

### Differences in the ALT Flap Thickness Between CT and Ultrasound Measurements

**Figure 4:**
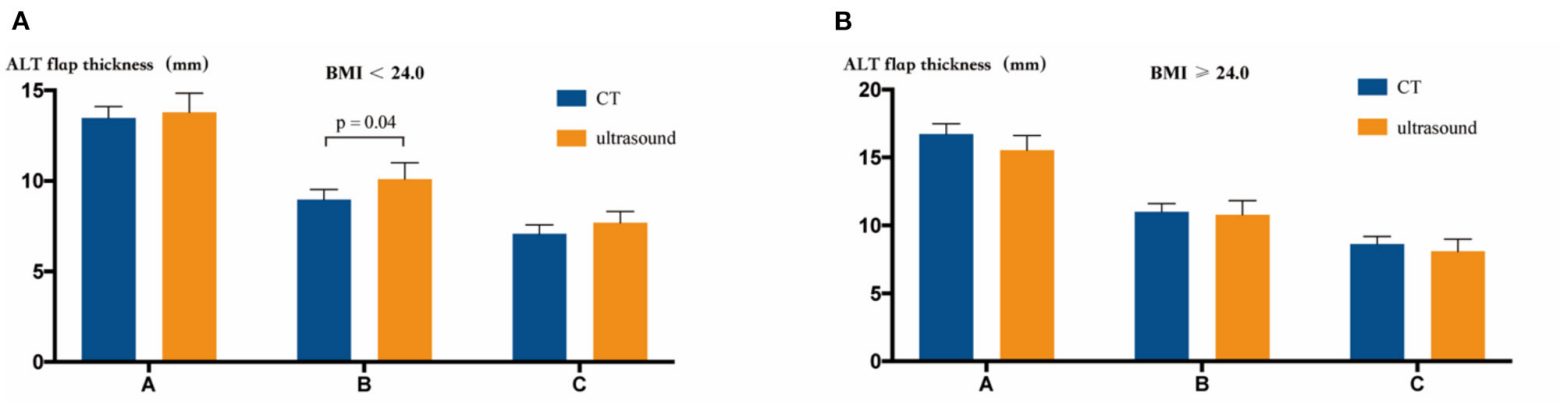
Comparison of CT vs. ultrasound measurement of the ALT flap thickness. **(A)** In the BMI < 24.0 group, CT and ultrasound measurements showed different results only at point B (*P* = 0.04, *t* test). **(B)** In the BMI ≥ 24.0 group, CT and ultrasound measurements showed no statistical differences at points A, B, and C.

We compared the differences in the ALT flap thickness between CT and ultrasound measurements. In the group with BMI < 24.0, only point B showed a statistically significant difference between the two measurements (8.96 mm and 10.10 mm in CT and ultrasound images, respectively, *P* = 0.04, *t* test). The group with BMI ≥ 24.0 showed no significant difference in the measured ALT flap thickness between CT and ultrasound (Figure 4).

### Correlation Between ALT Flap Thickness and BMI

**Figure 5:**
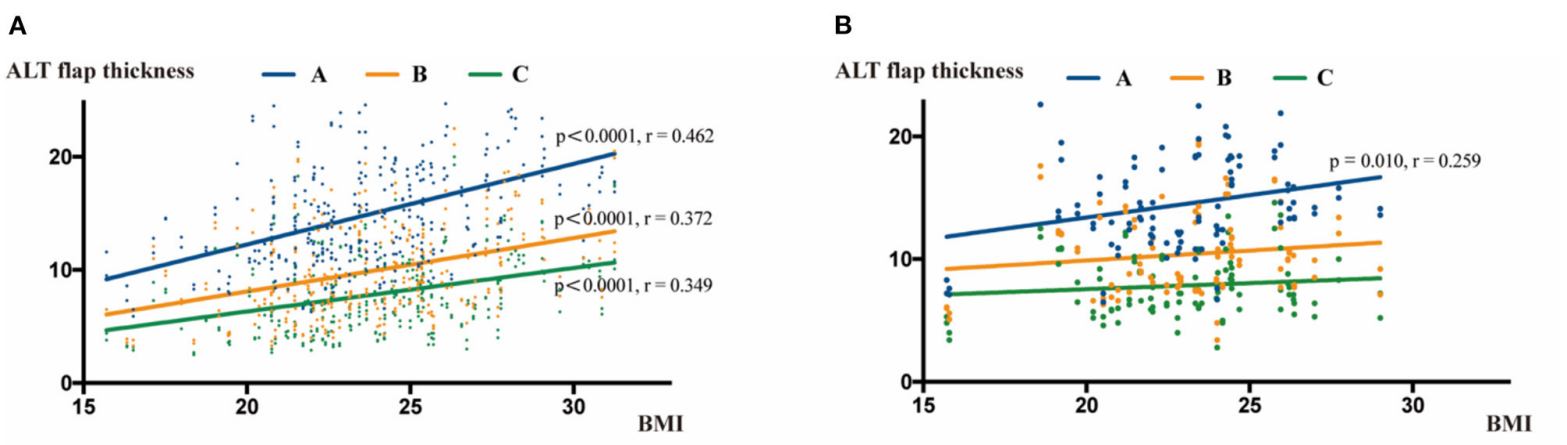
Correlation analysis of ALT flap thickness with BMI. **(A)** Correlation analysis between BMI and ALT flap thickness measured by CT scans. BMI was positively correlated with the thickness at points A, B, and C. **(B)** Correlation analysis between BMI and ALT flap thickness measured by ultrasound. BMI was positively correlated with the thickness only at point A.

We next tested the correlation between ALT flap thickness and BMI. During the CT measurement, the thicknesses at points A, B, and C were statistically significantly correlated with the BMI (*P* < 0.001, correlation analysis; r = 0.462, 0.372 and 0.349 at the points A, B and C, respectively, pearson test). During the ultrasound examination, a statistically significant correlation was only observed between BMI and the measurement at point A (*P* = 0.01, correlation analysis; r = 0.259, pearson test), but there was no correlation between BMI and measurements at points B and C (Figure 5).

## Discussion

By measuring the ALT flap thickness in individuals with different BMI values, our present study provided primary evidence to support a positive correlation between BMI and ALT flap thickness. With the development of microvascular anastomosis technology, grafting with a vascularized free flap has made it possible to repair and functionally restore tissue defects after maxillofacial trauma or tumor resection (15). Currently, the commonly used vascularized free flaps in the clinical practice include a radial forearm flap, deep inferior epigastric artery perforator flap, thoracodorsal artery perforator flap, deep circumflex iliac artery perforator flap, and ALT flap (16). Among them, the ALT flap has become the first choice to repair the head and neck, extremity, and truck defects in many medical centers (17, 18). With continuous advances in the microsurgical techniques, the survival rate of the ALT flap has been significantly improved. The incidence of donor complications has also been significantly reduced. The ALT flap has been used widely in the clinic, which makes it a *universal flap* in the clinical practice (19).

The thickness of the flap is particularly critical in the process of flap repair surgery. For example, in a patient with a tissue defect of the buccal mucosa near the mouth corner, a thick flap will cause difficulty in closing the mouth or limiting the mouth opening after surgery, whereas a thin flap can cause problems, such as deformed mouth corner, poor mouth closure, and excessive salivation. If the flap is too thick for a tissue defect of the soft palate, it will affect the patient's swallowing and pronunciation and obstruct the pharyngeal cavity, resulting in failure of removal of the tracheal tube on time post-operatively. For a soft tissue defect at the floor of the mouth, a thick flap could limit the tongue extension movement (20). Currently, selection of an ALT flap is mostly based on the clinician's experience. The thickness of an ALT flap varies significantly among different individuals. There is no reliable and objective method to select an appropriate ALT flap. In 2002 Nakayama et al. used ultrasound to measure the thickness of the radial forearm flap, ALT flap, and rectus abdominis flap in 31 patients (21). In 2004, Yu briefly described a correlation between the ALT flap thickness and BMI in 68 patients, and they found that the ALT flap thickness was significantly higher in women than in men (13). However, these studies had small sample sizes. In 2017, researchers in Taiwan applied ultrasound examination to analyze the correlation between BMI and the thickness of radial forearm flap, ALT flap, and lateral peroneal flap in an Asian population. The results showed that the radial forearm flap was thinner than the lateral peroneal flap, while the latter was thinner than the ALT flap. Among them, the ALT flap had the strongest correlation with BMI (22).

In the present study, we obtained accurate measurements of the ALT flap thickness based on the CT images of the lower extremity among study participants with different BMI values. Our results showed that the thicknesses at points A, B, and C, which were located on the line from the anterior superior iliac spine to the lateral superior patellar angle, decreased gradually. Meanwhile, at the same point, there was a significant difference in the ALT flap thickness between the two groups with BMI < 24.0 and BMI ≥ 24.0. The reason behind this difference night be that the thickness of the flap largely depends on the amount of subcutaneous fat. A high BMI is associated with an increased subcutaneous fat, which leads to an increase in the flap thickness. Since lower extremity images in patients undergoing ALT flap repair were often obtained by ultrasound rather than CT, we further evaluated 50 patients who underwent lower extremity ultrasound to study the differences in the flap thickness between patients with different BMI values. These results were compared with the results of the CT measurements. First, we found that the thicknesses at points A, B, and C located on the line from the anterior superior iliac spine to the lateral superior patellar angle also showed a decreasing trend under the ultrasound measurements. However, a statistically significant difference in the ALT flap thickness between patients with BMI < 24.0 vs. BMI ≥ 24.0 was only observed at point A (*P* = 0.03), which was consistent with the CT measurement results, but not at points B and C. On comparing the ultrasound and CT images, only point B showed a statistically significant difference in the BMI < 24.0 group (*P* = 0.04) between the two measurements. This suggested that ultrasound could replace CT to estimate the ALT flap thickness in a selected patient population.

In our correlation analysis, CT measurements could show a more significant correlation between the ALT flap thickness and BMI than ultrasound measurements. CT measurements could show significant correlations between the ALT flap thickness and BMI at points A, B, and C, whereas ultrasound measurements showed a significant correlation only at point A. We consider the following reasons for this occurrence: (1) CT scan was performed when patients were in a relaxed position. The data were obtained by the software. This could contribute to a more accurate measurement by CT images, and better reflect the true relationship between BMI and flap thickness. Ultrasound relies on the accuracy of the machine as well as the ultrasonographer, which can lead to more errors during the measurements; (2) We included fewer patients with ultrasound measurements in the retrospective analysis, which might be one of the reasons why no statistical significance was detected; (3) The ultrasonographer had to apply pressure on the probe during the ultrasound examination. The inconsistency in the pressure applied during the examinations could also contribute to measurement errors.

Limitations of the present study included a small sample size and single center research. Its retrospective design could have also introduced biases; thus, it has a lower reliability than randomized trials. The CT and ultrasound examinations were performed in different patients, which made it difficult to compare these two measurements. In addition, both the CT and ultrasound examinations are subjective. Future prospective research with a large sample size and more BMI categories can further clarify the relationship between the ALT flap thickness and BMI, which may provide better prediction of the ALT flap thickness and serve as a guide for the pre-operative estimation of the ALT flap thickness to achieve satisfactory surgery outcomes.

## Conclusion

There was a significant correlation between the ALT flap thickness and BMI. A higher BMI was correlated with a thicker ALT flap.

## Data Availability Statement

The raw data supporting the conclusions of this article will be made available by the authors, without undue reservation.

## Ethics Statement

We performed a retrospective study at the Ninth People's Hospital, Shanghai Jiaotong University School of Medicine, Shanghai, China. The study was approved by the Hospital Ethics Committee (SH9H-2021-T19-2). The requirement for obtaining informed consent from patients was waived. Written informed consent for participation was not required for this study in accordance with the national legislation and the institutional requirements.

## Author Contributions

PX and XQ conceived the study and revised the article. JY and LW did the literature research and collected clinical data. GY did the analysis of images. JY wrote the first draft of the article. All authors contributed to the article and approved the submitted version.

## Funding

This study was funded by the National Natural Science Foundation of China (No. 81971618).

## Conflict of Interest

The authors declare that the research was conducted in the absence of any commercial or financial relationships that could be construed as a potential conflict of interest.

## Publisher's Note

All claims expressed in this article are solely those of the authors and do not necessarily represent those of their affiliated organizations, or those of the publisher, the editors and the reviewers. Any product that may be evaluated in this article, or claim that may be made by its manufacturer, is not guaranteed or endorsed by the publisher.

## Abbreviations

ALT: anterolateral thigh
BMI: body mass index
CT: computed tomography.
